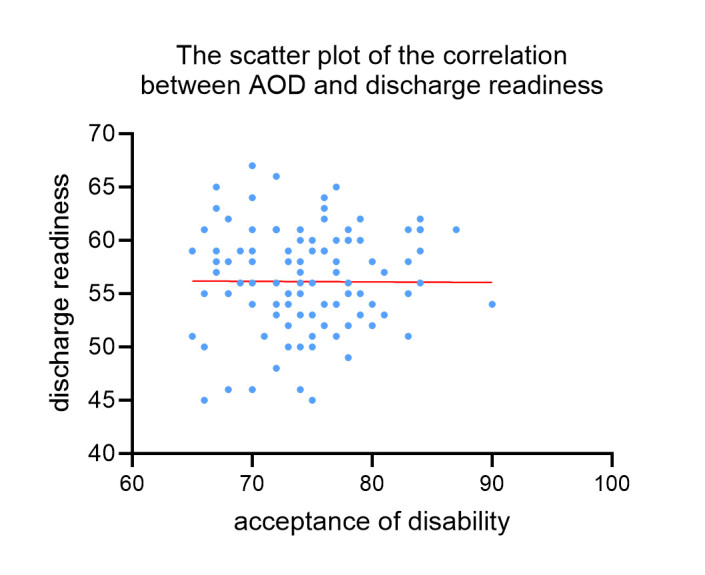# Acceptance of disability and discharge readiness in patients underwent modified radical mastectomy

**DOI:** 10.1097/MD.0000000000044047

**Published:** 2025-08-22

**Authors:** Man Zhu, Meijie Chen, Meng Wu, Shan Li, Yanyan Liao

**Affiliations:** a Department of Breast Surgery, The First Affiliated Hospital with Nanjing Medical University, Nanjing, Jiangsu, China.

**Keywords:** acceptance of disability, breast cancer, discharge readiness, modified radical mastectomy

## Abstract

This study investigates the acceptance of disability (AOD) and discharge readiness of patients after modified radical mastectomy and analyze the correlation between AOD and discharge readiness to provide scientific theoretical guidance for clinical practice. A total of 200 patients underwent modified radical mastectomy were evaluated by disability acceptance scale and discharge preparation scale. Single factor and multiple linear regression analysis were used to screen the influencing factors of AOD and discharge readiness of patients after modified radical mastectomy, and Pearson was used to analyze the correlation between AOD and discharge readiness. AOD score with 75.20 ± 9.16 and discharge readiness score with 56.30 ± 7.44 of patients after modified radical mastectomy were obtained. Multiple linear regression analysis showed that family economic status, whether spouses care about their current image, and whether undergo radiotherapy and chemotherapy were the influencing factors of AOD. Convenience of seeking medical treatment after discharge and education level were the influencing factors of discharge readiness score (*P* < .05). There is significant positive correlation between AOD and discharge readiness score (*P* < .05). Middle level of AOD and discharge readiness score of patients after modified radical mastectomy was reported and there is significant positive correlation between AOD and discharge readiness score, highlighting the importance of the help from medical staff on patients with breast cancer for their acceptance of breast loss after surgery, contributing to the enhancement of AOD and the quality of life.

## 1. Introduction

Breast cancer in China has increased to about 304,000 cases annually, which accounts for 24.2% of female cancers.^[1,2]^ At present, about 70% of breast cancer patients can be treated by mastectomy. Although the current surgical method for breast cancer has evolved from the classic radical breast cancer surgery to the modified radical breast cancer surgery, breast conserving surgery and breast reconstruction surgery for breast cancer, there are still some patients with breast loss due to disease duration, economic conditions, and other factors.^[3]^ However, neither traditional surgery nor modified radical mastectomy can avoid the loss of patients’ breasts, which will affect their external image, threaten their physical and mental health, and then go against postoperative rehabilitation.^[4]^ Due to the special physiological effects of the breasts, breast loss can easily lead to feelings of inferiority and even cause serious negative emotional reactions such as anxiety and depression. This physiological, psychological, anatomical, or functional abnormality or damage can be called disability.^[5]^

Research has shown that patients’ acceptance of disability (AOD) affects the degree of disease recovery and the quality of family life.^[6,7]^ AOD is a hot research topic in the field of psychology in recent years, which refers to the ability of individuals to correctly and profoundly recognize their own value and have the ability to cope with the negative effects of disability. Discharge readiness refers to the comprehensive evaluation by medical personnel of a patient’s physical, psychological, and social health status, analyzing and judging their level of ability to leave the hospital, return to society, further recovery.^[8,9]^ After modified radical mastectomy, patients need to face various problems in wound management, adverse reactions from follow-up treatment, functional exercise of affected limbs, and other difficulties.^[10,11]^ The risk of complications tends to increase if they leave the hospital without sufficient preparation. Therefore, discharge preparation plays an important role in promoting the rehabilitation of patients taking modified radical mastectomy. Nevertheless, there are few research reports about AOD and discharge readiness of patients after modified radical mastectomy.

Based on this, this paper aims to investigate the current status of AOD and discharge readiness of patients who underwent modified radical mastectomy, and analyze the correlation between AOD and discharge readiness to provide theoretical basis for patients to develop standardized discharge guidance.

## 2. Methods

### 2.1. Study design and participants

This study was designed as a prospective, cross-sectional clinical study. Two hundred cases of patients underwent modified radical mastectomy from January 1st, 2020 to June 1st, 2023 were selected as the research objects. Inclusion criteria were as follows: patients were diagnosed as breast cancer by pathological examination; age ≥ 18 years old; patients underwent modified radical mastectomy in our center; patients voluntarily participate in the study. Exclusion criteria were: estimated survival time <6 months; not cooperating with the research or having difficulty understanding the questionnaire survey; unable to complete the survey due to language barriers, unclear consciousness, and other reasons; accompanied by other malignant tumor diseases; incomplete clinical data. The protocols used in this study were approved by the local ethics committee of the First Affiliated Hospital of Nanjing Medical University (2022-SRFA-429). Written informed consent was obtained from all the enrolled patients.

### 2.2. Sample size calculation

Sample size calculation was performed using a single population proportion formula. To ensure the precision of our results, we used this proportion with a 95% confidence level and a 5% margin of error, resulting in an initial sample size calculation of 200.

### 2.3. Procedure of modified radical mastectomy

The patient is well-positioned supine and a curvilinear or elliptical incision is made on the breast, typically encompassing the tumor or previous biopsy site. Skin flaps are carefully elevated and breast tissue, including the nipple–areolar complex, is removed in one piece. The axilla is opened, and levels I and II axillary lymph nodes are identified and removed. Hemostasis is achieved using electrocautery or ligation of blood vessels to control bleeding. Meticulous attention is paid to ensure no residual bleeding in the axilla and chest wall. Surgical drains are inserted and the skin flaps are repositioned with the incision closed in layers.

### 2.4. Research methods

#### 2.4.1. General information survey form

The content structure is independently designed, mainly including age, education level, marital status, work situation, family economic status, whether it is convenient to seek medical treatment after discharge, whether chemotherapy is used, residential situation, convenient medical treatment after discharge, and whether the spouse cares about the current image.

#### 2.4.2. Acceptance of Disability Scale

The scale form was used to describe an individual’s attitude towards disability.^[12]^ In this study, coefficient of Cronbach α is 0.83. The scale has 4 dimensions and 32 items, including 9 items for expansion, 9 items for transformation, 9 items for inclusion, and 5 items for dependent attributes. Each item is assigned a score of 1 to 4 points, with a total score of 32 to 128 points. Patients with higher score would be considered as higher level of AOD.

#### 2.4.3. Readiness for Hospital Discharge Scale

In this study, coefficient of Cronbach α is 0.83. There are a total of 3 dimensions and 12 items, including personal status (3 items), expected support (4 items), and adaptability (5 items). Each item is scored on a scale of 0 to 10, with a total score of 0 to 120 points. Patients with higher score would be identified with better readiness for discharge.

### 2.5. Investigation methods

Enrolled patients who underwent modified radical mastectomy completed the questionnaire within 4 hours before discharge. The investigators used unified guidelines to explain the purpose and significance of the survey. All questionnaires should be completed by patients themselves without disturbance. Data was verified and input by 2 independent staff in our center. A total of 210 questionnaires were distributed in this study, 206 of which were collected. Regular questionnaires, inconsistencies or consistency of options were excluded, resulting in 200 valid questionnaires.

### 2.6. Statistical analysis

SPSS software (SPSS Inc., Chicago) was used to input and analyze data. Measurement data that conforms to a normal distribution (mean ± standard deviation) was analyzed using *t* test. Count data was described as n (%) and analyzed by analysis of variance (ANOVA). Multiple linear regression analysis was used to identify the influencing factors of AOD and discharge readiness. Pearson analysis was used to examine the correlation between AOD and discharge readiness. *P* < .05 was considered as statistically significant difference.

## 3. Results

### 3.1. Basic characteristics of patients

Basic characteristics of patients were presented in Table 1. All patients enrolled in this study are females, with an age ranging from 43 to 67 years old and an average age of (50.46 ± 3.19) years. Among them, 108 patients were with tumor node metastasis classification stage I and 92 with tumor node metastasis classification stage II. Moreover, there were 61 patients with primary school education or below, 57 patients with junior high school education, and 82 patients with high school education or above.

**Table 1:** Basic characteristics of enrolled patients.

Variables	Numbers
Age (yr; mean ± SD)	50.46 ± 3.19
≥60 (n; %)	41 (20.5)
45–59 (n; %)	76 (38.0)
<45 (n; %)	83 (41.5)
TNM stage	
Stage I (n; %)	108 (54.0)
Stage II (n; %)	92 (46.0)
Education	
Primary or below (n; %)	61 (30.5)
Junior high (n; %)	57 (28.5)
High school and above (n; %)	82 (41.0)
Economic status	
Income higher than expense (n; %)	46 (23.0)
Balance of payment (n; %)	94 (47.0)
Income lower than expense (n; %)	60 (30.0)
Marriage	
Married (n; %)	154 (77.0)
Single/divorced (n; %)	46 (23.0)
Employment	
Employed (n; %)	97 (48.5)
Unemployed (n; %)	55 (27.5)
Self-employed (n; %)	48 (24.0)
Convenience of medication after discharge	
Yes (n; %)	115 (57.5)
No (n; %)	85 (42.5)
Chemotherapy/radiotherapy	
Yes (n; %)	83 (41.5)
No (n; %)	117 (58.5)
Present appearance cared by spouses	
Yes (n; %)	108 (54.0)
No (n; %)	92 (46.0)

TNM = tumor node metastasis classification.

### 3.2. AOD and discharge readiness scores of patients after modified radical mastectomy

AOD score and discharge readiness score of patients after modified radical mastectomy are shown in Table 2. For the subgroup analysis based on basic characteristics, significant difference were observed in AOD scores of patients with modified breast cancer in subgroup of economic status, Chemotherapy/radiotherapy, and present appearance cared by spouses (*P* < .05; Table 3). In addition, there were statistical difference in discharge readiness of patients from subgroups of convenience of medication after discharge and education (*P* < .05; Table 3). Subsequently, significant difference between AOD and discharge readiness was identified in the subgroup of present appearance cared by spouses (*P* = .029), while no difference in other subgroup analysis (*P* > .05).

**Table 2:** AOD and discharge readiness scores of patients after modified breast cancer surgery.

Variables	Number of entries	Score range	Score (mean ± SD)	Average score of entries (mean ± SD)
AOD	32	32–128 points	75.20 ± 9.16	2.35 ± 0.33
Expansion	9		22.03 ± 3.36	2.45 ± 0.35
Change	9	9–36 points	23.40 ± 3.38	2.60 ± 0.42
Inclusive	9	9–36 points	17.63 ± 2.07	1.96 ± 0.20
From attributes	5	5–20 points	12.14 ± 2.25	2.43 ± 0.38
Discharge readiness	12	0–120 points	56.30 ± 7.44	4.69 ± 0.73
Personal status	3	0–30 points	12.59 ± 2.30	4.20 ± 0.66
Expected support	4	0–40 points	20.18 ± 3.11	5.05 ± 0.71
Adaptability	5	0–50 points	23.53 ± 3.26	4.71 ± 0.57

AOD = acceptance of disability; SD = standard deviation.

**Table 3:** Comparison of AOD and discharge readiness of patients after modified breast cancer surgery.

Variables	AOD	*T*/*F* value	*P*-value	Discharge readiness	*T*/*F* value	*P*-value
Age (years; mean ± SD)		1.62	.25		3.06	.31
≥60	76.02 ± 9.25			58.11 ± 7.36		
45–59	74.85 ± 9.28			56.83 ± 7.50		
<45	73.55 ± 8.18			55.45 ± 7.42		
Education		1.26	.62		50.19	<.001
Primary or below	75.22 ± 9.45			61.43 ± 9.68		
Junior high	73.82 ± 9.06			52.53 ± 6.17		
High school and above	71.94 ± 9.17			47.58 ± 5.66		
Marriage		2.48	.19		0.95	.37
Married	72.27 ± 9.35			55.51 ± 7.04		
Single/divorced	74.93 ± 9.36			56.80 ± 7.22		
Employment		2.51	.10		2.73	.094
Employed	72.06 ± 9.35			54.91 ± 6.77		
Unemployed	75.45 ± 9.53			57.79 ± 7.80		
Self-employed	74.73 ± 9.55			55.65 ± 7.33		
Economic status		12.38	<.001		1.47	.18
Income higher than expense	80.74 ± 9.81			57.46 ± 8.75		
Balance of payment	75.43 ± 936			56.45 ± 7.59		
Income lower than expense	70.54 ± 9.11			54.43 ± 7.30		
Convenience of medication after discharge		3.36	.21		7.11	<.001
Yes	75.32 ± 9.28			52.50 ± 6.93		
No	73.22 ± 9.35			58.40 ± 7.39		
Chemotherapy/radiotherapy		7.09	<.001		0.71	.52
Yes (n; %)	68.37 ± 8.52			55.30 ± 7.17		
No (n; %)	75.93 ± 9.50			56.08 ± 7.29		
Present appearance cared by spouses		6.38	<.001		3.16	.048
Yes (n; %)	67.31 ± 7.40			54.88 ± 7.30		
No (n; %)	75.83 ± 9.80			57.16 ± 7.53		

AOD = acceptance of disability, SD = standard deviation.

### 3.3. Multiple linear regression analysis of AOD and discharge readiness of patients after modified radical mastectomy

Using AOD as the dependent variable, the independent variables are statistically significant single factor factors (family economic status, whether radiation and chemotherapy are performed, and whether spouse cares about current image), as shown in Table 4. The results showed that economic status, present appearance cared by spouses, and chemotherapy/radiotherapy were identified to be the influencing factors of AOD (*P* < .05; Table 4). The results of multiple linear regression showed that the convenience of medication after discharge and the level of education were the influencing factors of patient readiness for discharge (*P* < .05; Table 5). Furthermore, no significant difference was observed among these influencing factors (*P* > .05).

**Table 4:** Multiple linear regression analysis of AOD in patients.

Variables	*B*	*SE*	*P* value
Economic status	-4.63	1.49	.001
Chemotherapy/radiotherapy	2.25	0.62	<.001
Present appearance cared by spouses	2.12	0.41	<.001

AOD = acceptance of disability.

**Table 5:** Multiple linear regression analysis of discharge readiness in patients.

Variables	*B*	*SE*	*P* value
Education	-3.89	0.76	<.001
Convenience of medication after discharge	2.14	0.23	<.001

### 3.4. Correlation analysis between AOD and discharge readiness of patients after modified radical mastectomy

Subsequently, we performed the correlation analysis between AOD and discharge readiness of patients underwent modified radical mastectomy. As a result, there is significantly positive correlation between AOD and discharge readiness (*R* = 0.225–0.941, *P* < .05; Table S1, Supplemental Digital Content, https://links.lww.com/MD/P753 and Fig. S1, Supplemental Digital Content, https://links.lww.com/MD/P752).

## 4. Discussion

It is necessary for patients underwent modified radical mastectomy to face various problems in wound management, adverse reactions, and discharge appearance. In this study, we have explored the effect of AOD and discharge readiness on patients taking modified radical mastectomy. It is indicated that various factors contributed to AOD and discharge readiness in patients before discharge from hospital, and more importantly, AOD is significantly associated with discharge readiness, highlighting the combination application of AOD and discharge readiness in clinical nursing and rehabilitation.

After mastectomy of breast cancer, patients not only face anatomical or functional damage, but the loss of secondary characteristic breasts can cause serious psychological pressure, reduce their family and social adaptability, and affect their quality of life.^[13]^ AOD refers to an individual’s acceptance of their disability status.^[14]^ It plays an important role in promoting health outcomes and is particularly crucial in mediating the physical and mental health and life satisfaction of patients.^[15]^ Most patients are often difficult to accept their disability status, and a series of negative emotions are generated. On the 1 hand, mastectomy brings irreversible damage and permanent defects to the patient’s body, reduces their sense of self-identity. Long-term treatment exacerbates the financial burden on families, making it easier for patients to feel guilty and self-blame.^[16]^ In addition, insufficient understanding of disease-related knowledge is not conducive to establishing a correct understanding of one’s own disability status. Recent progress in the acceptance of disability has focused on the development and implementation of effective interventions. Acceptance and commitment therapy has shown promise in helping individuals with disabilities to accept their conditions and improve their psychological well-being.^[17]^ Research has also highlighted the importance of interventions that enhance self-satisfaction and promote the meaning-making process, which can lead to the development of new goals and better coping skills. Furthermore, interventions are being extended to family members of individuals with disabilities to provide emotional support and increase awareness of the impact of disability on the family.^[18]^

It is found that family economic status was the influencing factors for improving AOD of postoperative patients with breast cancer. Several studies have found that the better the family economic status, the higher the patient’s AOD, which is consistent with this study.^[19,20]^ Related treatments have brought serious economic burden to families, and patients with low income levels may experience feelings of self-blame, guilt, and other psychological factors, making it difficult for them to objectively recognize disability. It is recommended that clinical medical staff should fully evaluate different types of family status and assist patients in finding resources from their families.

Furthermore, this article found that patients who undergo radiotherapy and chemotherapy have lower AOD. The root cause is that radiation therapy can damage the skin, while chemotherapy can cause hair loss and affect personal image. It is also reported that patients whose spouse cares about their image have lower AOD, which can be explained by that some patients believe that they have defects that affect their marital life, and even develop complex psychological states which can affect AOD.^[21]^ It is recommended that clinical medical staff instruct family members to unite and view the disease as a shared responsibility of the entire family during health education.

The score of discharge readiness in this study is at the lower middle level, lower than the score of discharge readiness of breast cancer patients study, indicating that the improved discharge readiness of patients after breast cancer surgery needs to be further strengthened.^[22]^ Possible factors that may be related to pre discharge pain or discomfort, breast loss caused by surgery, and subsequent treatment leading to negative emotions in patients after discharge. Moreover, there is significant positive correlation between AOD and discharge readiness. It shows that patients with high level of disability acceptance can quickly accept the fact of disability with the second sex sign, give themselves good psychological hints, establish good postoperative recovery confidence and are more willing to cooperate with the hospital process.^[23]^ For this type of patient, it is more conducive to establishing correct self-awareness, seeing valuable and meaningful parts beyond disability.^[24]^ For patients with lower acceptance of disability, they may immerse themselves in the emotional weakening of femininity caused by breast loss, contributing to lower readiness.

In addition, we identified that education level and whether it is convenient to go to hospital after discharge were the influencing factors of the improved breast cancer patients’ discharge readiness. Previous studies have reported that patients with a high level of education may have more ways to acquire disease-related knowledge and health management skills, which can enhance their understanding of discharge education content.^[21,25]^ Low educated patients limit their ability to receive information and utilize medical resources, resulting in lower readiness for discharge. It is suggested that clinical doctors and nurses can provide follow-up visits for patients who are not able to get medical treatment.

To sum up, AOD and discharge readiness scores of patients after modified radical mastectomy are at a moderate level, of which significantly positive correlation was observed. It is suggested that medical staff should pay more attention on the improvement of the postoperative patients with breast cancer to establish correct cognition and optimistic acceptance of the fact of breast loss, enhance their AOD, so as to improve their preparation for discharge and improve their quality of life. However, this study is only a cross-sectional study, without longitudinal attention to the impact of AOD on the discharge readiness of patients after modified radical mastectomy, and will continue to follow up in the future.

## Acknowledgments

We would like to thank all the participants of this project.

## Author contributions

**Conceptualization:** Man Zhu, Yanyan Liao.

**Data curation:** Man Zhu, Meijie Chen.

**Formal analysis:** Meijie Chen, Yanyan Liao.

**Funding acquisition:** Yanyan Liao.

**Investigation:** Man Zhu.

**Methodology:** Meijie Chen, Meng Wu.

**Project administration:** Yanyan Liao.

**Resources:** Meijie Chen.

**Software:** Meng Wu, Shan Li.

**Supervision:** Meng Wu, Shan Li.

**Validation:** Shan Li.

**Writing – original draft:** Man Zhu.

**Writing – review & editing:** Yanyan Liao.

## Supplementary Material



**Figure:**