# Supplementary material for: Acceptance of disability and discharge readiness in patients underwent modified radical mastectomy
Source: Medicine (Baltimore). 2025 Aug 22;104(34):e44047. doi: 10.1097/MD.0000000000044047 (PMC12384890; doi:10.1097/MD.0000000000044047)
Supplement: Supplementary file 1 [file medi-104-e44047-s001.docx]

**Supplemental Table 1. Correlation analysis between AOD and discharge readiness**

| **Variable** | **Discharge readiness** | **Personal status** | **Expected support** | **Adaptability** |
| --- | --- | --- | --- | --- |
| **AOD** | 0.941^**^ | 0.815^**^ | 0.530^**^ | 0.643^**^ |
| **Expansion** | 0.556^**^ | 0.232^*^ | 0.257^*^ | 0.720^**^ |
| **Change** | 0.739^**^ | 0.704^**^ | 0.784^**^ | 0.225^*^ |
| **Inclusive** |  | 0.753^**^ | 0.565^*^ | 0.756^**^ |
| **From attributes** | 0.771^**^ | 0.576^**^ | 0.616^**^ | 0.539^**^ |

* *P*<0.05;** *P*<0.01

Abbreviations: AOD, acceptance of disability.
